# Mint-Scented Species in Lamiaceae: An Abundant and Varied Reservoir of Phenolic and Volatile Compounds

**DOI:** 10.3390/foods13121857

**Published:** 2024-06-13

**Authors:** Tilen Zamljen, Mariana Cecilia Grohar, Aljaz Medic

**Affiliations:** Department of Agronomy, Biotechnical Faculty, University of Ljubljana, Jamnikarjeva 101, SI-1000 Ljubljana, Slovenia; marianacecilia.grohar@bf.uni-lj.si (M.C.G.); aljaz.medic@bf.uni-lj.si (A.M.)

**Keywords:** cultivars, metabolomics, mint, HPLC/MS, volatiles, phenolics

## Abstract

This investigation aimed to identify the most favorable cultivar based on plant metabolites for potential targeted cultivation in the pharmaceutical industry. The analysis revealed the presence of 19 individual phenolics and 80 individual volatiles across the cultivars, a breadth of data not previously explored to such an extent. Flavones emerged as the predominant phenolic group in all mint-scented cultivars, except for peppermint, where hydroxycinnamic acids dominated. Peppermint exhibited high concentrations of phenolic acids, particularly caffeic acid derivatives and rosmarinic acid, which are known for their anti-inflammatory and antioxidant properties. Luteolin-rich concentrations were found in several mint varieties, known for their antioxidative, antitumor, and cardio-protective properties. Swiss mint and spearmint stood out with elevated levels of flavanones, particularly eriocitrin, akin to citrus fruits. Monoterpene volatiles, including menthol, camphor, limonene, and carvone, were identified across all cultivars, with Swiss mint and spearmint exhibiting the highest amounts. The study underscores the potential for targeted cultivation to enhance volatile yields and reduce agricultural land use. Notably, chocolate mint demonstrated promise for volatile content, while apple mint excelled in phenolics, suggesting their potential for broader agricultural, pharmaceutical, and food industry production.

## 1. Introduction

Mint-scented (named mints in manuscript) species such as *Mentha* and *Nepeta* belong to the Lamiaceae family and are extensively cultivated across Europe, Asia, Africa, Australia, and North America, with at least 42 recorded species and 15 hybrids [1]. Primarily perennial, these plants are valued for their abundant essential oil content, with economically significant species including *Mentha aquatica* L., *Mentha canadensis* L., *Mentha spicata* L. (spearmint), and notable hybrids like *Mentha* × *piperita* L. (peppermint) [2]. The versatile applications of mints encompass herbal teas, spices, confectionery, toothpaste, chewing gum, beverages, bakery products, cosmetics, oral hygiene items, pharmaceuticals, and pesticides [3]. Throughout history, mint leaves, flowers, and stems have been integral to herbal teas and spices for their aromatic and flavorful qualities [4]. Traditional medicine has employed mints for various purposes, including alleviating headaches, fever, digestive issues, and minor ailments. Modern medicine acknowledges the utility of mint species, particularly in addressing gastrointestinal disorders [4]. Clinical studies support the anti-inflammatory properties of mint essential oils, with *M. spicata* essential oil demonstrating analgesic effects attributed to key components such as carvone, limonene, and menthol [5].

The diverse mint species exhibit varied growth requirements. While some, like *M. arvensis*, thrive in tropical and sub-tropical conditions (20–25 °C), others, like peppermint and spearmint, prefer cool to temperate regions, necessitating long days with higher temperatures (21 °C to 26 °C) and cool nights for optimal essential oil compound balance [2]. Mint cultivation is generally less demanding regarding soil types, with pH playing a crucial role, ideally ranging from 6 to 7.5. Adequate irrigation, especially in higher temperatures, is vital for successful cultivation [6,7].

Mints are renowned for their rich plant metabolites, particularly volatile compounds. The most common mint volatiles are menthol, menthone, menthyl acetate, 1,8-cineole, menthofuran, isomenthone, neomenthol, and limonene [2]. Volatiles represent one of the largest groups of metabolites in plants. They are usually lipophilic liquids with high vapor pressures [8]. Volatiles, comprising terpenoids, alcohols, esters, and aldehydes, serve various functions, such as attracting pollinators, protecting plant generative parts against pathogens, parasites, and herbivores, and participating in inter-plant and intra-plant signaling [9,10]. Monoterpenoids like eucalyptol, (±)camphorquinone, and menthol are common in mints and find applications in agricultural, pharmaceutical, and food industries as insecticides, antiallergenic agents, perfumes, and food additives [1,11]. Mints also contain a wealth of phenolic substances, a crucial group of compounds responsible for the antioxidant activity observed in mint [12]. These compounds act as a protective shield for plants against UV radiation and attacks from pests and diseases. According to Brown, et al. [13], the total polyphenolic content in peppermint leaves is approximately 19–23%, with total flavonoids constituting 12%. Phenolic compounds play a pivotal role in promoting human health by mitigating the adverse effects of oxidative stress on the body [14].

Despite the widespread use of mint-derived substances, the current reliance on natural resources for their extraction poses environmental challenges due to the need for extensive agricultural land and environmentally costly, low-yielding extraction processes [15]. Given the diverse array of plant metabolites found in mints and the growing demand for health-promoting foods, studies focusing on mints and similar herbs hold significant importance. In our study, we investigated 11 mint cultivars (10 from *Mentha* species and 1 *Nepeta* species), which are the most economically relevant in Slovenia. All species are considered mints and have a mint scent, so cat mint was also part of the study. We identified 19 individual phenolics and 80 individual volatile compounds. The main aim of our study was to determine the optimal mint cultivar in terms of metabolism for a more targeted cultivation approach. This analysis is fundamental for the pharmaceutical industry to harvest the best phenolic/volatile-rich mints. This approach could potentially decrease the need for agricultural land and yield higher metabolite contents in industrial extraction processes. Additionally, the consumption of the most phenolic and volatile-rich mint cultivars could result in better health-promoting effects in humans.

## 2. Materials and Methods

Mint plants were planted at the Biotechnical Faculty, Ljubljana (46°3′4″ N; 14°30′18″ E). The eleven most economically important mint-scented cultivars in Slovenia were grown as a part of a herbs collection. The cultivars were as follows: peppermint (*Mentha* × *piperita*), Swiss mint (*Mentha* × *piperita* ‘Swiss’), chocolate mint (*Mentha* × *piperita* var. *citrata*), apple mint (*Mentha suaveolens*), strawberry mint (*Mentha spicata* var. *citrata*), spearmint (*Mentha spicata*), lemon mint (*Mentha* × *piperita* var. *citrate*), Moroccan mint (*Mentha spicata* var. *crispa*), cat mint (*Nepeta cataria*), orange mint (*Mentha* × *piperita* var. *citrate*), and English peppermint (*Mentha piperita* L.). Cat mint was chosen to be part of the study because of its mint-like appearance, nomenclature, and mint scent, even though it is a different species (cat mint and other mints are referred to throughout the manuscript with the common name “mints”). The plants were grown using the principles of good agricultural practice, with constant irrigation and fertilization based on the guidelines of the Ministry of Agriculture, Forestry, and Food [16].

### 2.1. Plant Material Sampling

The samples were collected when the mint plants were ready for harvest, in our case, on 12 July 2023. The leaves and stems were cut using scissors and placed into liquid nitrogen. The samples were then transported into the laboratory at the chair for fruit science, where they were ground with a mortar to a fine powder and stored at −20 °C.

### 2.2. Extraction of Phenolic Substances

The extraction of phenolic was based on the methodology of Zamljen, et al. [17]. In brief, 0.1 g of fresh mint leaves was cut, ground to fine dust, and extracted with 4 mL of 70% methanol and 3% formic acid in bi-distilled water using a pestle and mortar. The entire extraction procedure was carried out at 0 °C to 4 °C to prevent oxidation. The samples were then placed into an ultrasonic bath (Sonis 4 ultrasonic bath; Iskra pio, Sentjernej, Slovenia, working at 30 kHZ and 400 W) for 1 h (0 °C) and, after sonification, centrifuged at 8000× *g* for 5 min and filtered through a 0.25 µm polyamide filter (Chromafil AO-20/25, Macherey-Nagel, Dueren, Germany). The samples were then stored in 1.5 mL vials at −20 °C.

### 2.3. Identification and Quantification of Phenolic Substances

The induvial phenolic substances were identified using tandem mass spectrometry (MS/MS; LTQ XL; Thermo Scientific, Waltham, MA, USA) in negative ion mode. The quantification was carried out on a UHPLC system (Vanquish; Thermo Scientific, Waltham, MA, USA). Further HPLC/MS parameters were previously described by Zamljen, et al. [17].

For the calculation of the individual phenolics quantity, their respective standards were used. Where the standards were not available, phenolics were expressed as equivalents of a similar substance.

All luteolins were calculated based on luteolin-3-glucoside standard and expressed as equivalents. Eriocitrin and diosmin were expressed as naringenin equivalents. Lithospermic acid derivative and salvianolic acid were expressed as caffeic acid equivalents. Rosmarinic acid, salvianolic acid, salvianolic acid A isomer, salvianolic acid B isomer, rosmarinic acid derivative, salvianolic acid B/E isomer, and sagerinic acid were expressed as *p*-coumaric acid equivalents. The data of individual phenolics are presented as mg/100 g FW.

### 2.4. Identification and Semi-Quantification of Volatile Compounds

The identification and semi-quantification of volatile compounds was carried out based on the methodology by Zamljen, et al. [18]. In brief, 0.3 g of ground leaves was placed into 20 mL glass vials. The entire extraction process was carried out at room temperature at 21 °C. The analysis was carried out on a Shimadzu GC-MS QP2020 gas chromatograph connected with a Single Quadropole MS with an EI detector. Further apparatus details were previously described in detail by Zamljen, et al. [17]. The volatiles were identified based on their retention indices (RIs) and commercial libraries of spectra (NIST 11 and FFNSC 4). The semi-quantification was based on each compound and the internal standard peak areas, taking into consideration the internal standard and sample weight. The volatile contents were expressed as mg/100 g FW.

### 2.5. Chemicals

The standards used in the experiment were as follows: caffeic acid, *p*-coumaric acid, naringenin, luteolin-3-glucoside, and 3-nonanone (Sigma–Aldrich Chemie GmbH, Steinheim, Germany).

### 2.6. Statistical Analysis

The R program was used for statistical analysis. All data are expressed as mean ± standard error (SE). To determine whether differences were observed between treatments, a one-way analysis of variance (ANOVA) with a Tukey test was used. The confidence level was 95%.

## 3. Results

### 3.1. Identification of Individual Phenolics in Mint Cultivars

In the eleven mint cultivars, 19 individual phenolics were identified, of which there were 11 hydroxycinnamic acids, 6 flavones, and 3 flavanones (Table 1). Among the 11 hydroxycinnamic acids, protocatechuic acid was identified by the typical fragmentation pattern of MS^n^ *m*/*z* 153 and caffeic acid by its typical fragmentation pattern of MS^n^ *m*/*z* 179, 161, and 133, as reported by Imen Belhadj, et al. [19]. Rosmarinic acid and rosmarinic acid derivatives were identified by their typical fragmentation pattern of MS^n^ *m*/*z* 161, 179, 197, and 223, as reported by Xu, et al. [20]. Salvianolic acid and three isomers were identified in mints by their fragmentation pattern of MS^n^ *m*/*z* 339, 519, 537, and 493, as reported by Zhu, et al. [21]. Lithosperimic acid derivative was identified by its characteristically typical fragmentation pattern of MS^n^ *m*/*z* 359 and 295, as reported by Grzegorczyk-Karolak, et al. [22]. Sagerinic acid was identified based on its mass *m*/*z* 719 and a typical fragmentation of MS^2^ *m*/*z* 557 and 359 and MS^3^ *m*/*z* 359, 161, 179, 197, and 223, as reported by Serrano, et al. [23].

The six flavones were all identified by the typical fragmentation pattern of luteolin MS^n^ *m*/*z* 447, 461, and 285, as reported by Zamljen, et al. [24]. The three flavanones were eriocitrin, diosmin, and naringenin-7-*O*-rutinoside. Eriocitrin was identified by its molecular mass of *m*/*z* 595.5 and fragmentation pattern of MS^2^ *m*/*z* 287, as reported by Li, et al. [25]. Diosmin was identified by its typical fragmentation pattern of MS^n^ *m*/*z* 299 and 284, as reported by Wang, et al. [26]. Naringenin-7-*O*-rutinoside was identified by its typical identification pattern of MS^n^ *m*/*z* 271 and 270, as reported by Lee, et al. [27].

### 3.2. Individual and Total Phenolics Contents in Eleven Mint Cultivars

**Figure 1:**
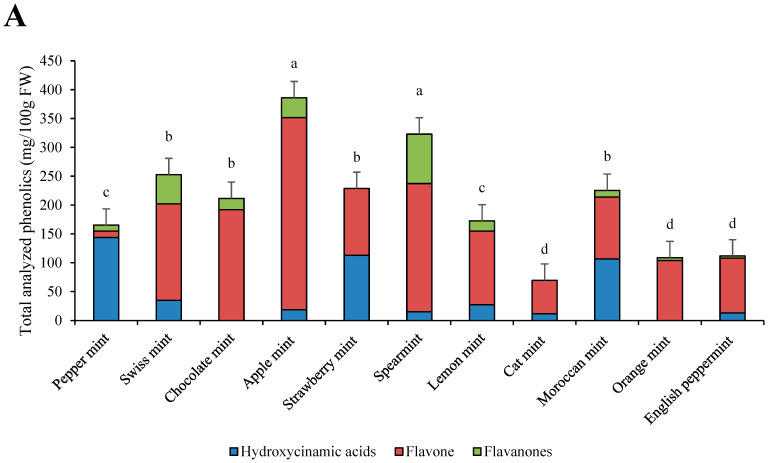

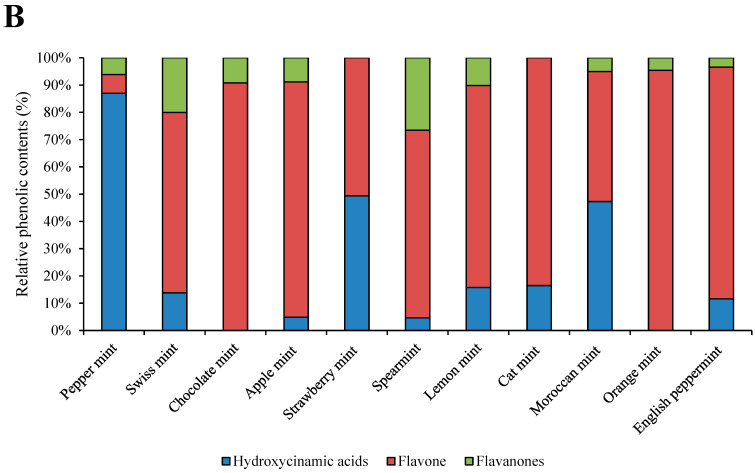
Individual phenolic groups and total analyzed phenolic contents in eleven mint cultivars (**A**) and their relative contents (**B**). a–d: statistical significant differences among different mint scented cultivars.

Total analyzed phenolics and their relative contents are presented in Figure 1A,B. Great variability was shown among the phenolic contents of mints. The most phenolics were present in apple mint and spearmint, with 385.80 mg/100 g FW and 322.98 mg/100 g FW, respectively. The lowest contents of phenolics were in cat mint, with 69.56 mg/100 g FW. The most abundant hydroxycinnamic acid was lithosperimic acid derivative, with especially high concentrations in strawberry mint (112.90 mg/100 g FW) (Table 2). The most abundant flavone was luteolin rutinoside, with the highest concentration in apple mint (180.02 mg/100 g FW). The most abundant flavanone was eriocitrin, especially in spearmint (85.76 mg/100 g FW). Cat mint was the only species that contained protocatehuic acid (8.62 mg/100 g FW). Rosmarinic acid, salvianolic acid A isomer, salvianolic acid B isomer, and salvianolic acid B/E isomer were only found in peppermint and no other mint cultivar. Moroccan mint had, as the only mint cultivar, high amounts of sagerinic acid, with 60.70 mg/100 g FW.

The most abundant phenolics were flavones in all cultivars except in peppermint, in which 85% of the total analyzed phenolics were hydroxycinnamic acids. In strawberry mint and cat mint, no flavanones were identified, and in chocolate mint and orange mint, no hydroxycinnamic acids were identified.

### 3.3. Volatile Organic Compounds in Different Mints

#### Identification of Individual Volatiles in Mint Cultivars

In Table 3, the identification of 80 individual volatiles is detailed, as well as the cultivar in which each volatile was present. In general, 30 (apple mint) to 44 (English peppermint) individual volatiles were identified (Table 3). Certain volatiles were present only in individual mint cultivars. In orange mint, two specific volatiles were found, namely lavandulol and geraniol. Bornyl acetate was only found in peppermint. *Cis*-pinocamphone was identified only in spearmint. Strawberry mint, as the only analyzed mint cultivar, contained myrtenal, *p*-mentha-1,8-dien-3-one, and *cis*-jasmone. In English peppermint, one specific volatile was identified, namely neomenthyl acetate. Lemon mint contained a specific volatile named vaporole. Moroccan mint contained three specific volatiles, namely *p*-mentha-1,3,8-triene, sabinene hydrate, and *cis*-3-hexenyl-isovalerate. Cat mint contained the most specific volatiles (5), namely styrene, copaene, *β*-bisabolene, nepetalactone 1, and nepetalactone 2.

**Figure 2:**
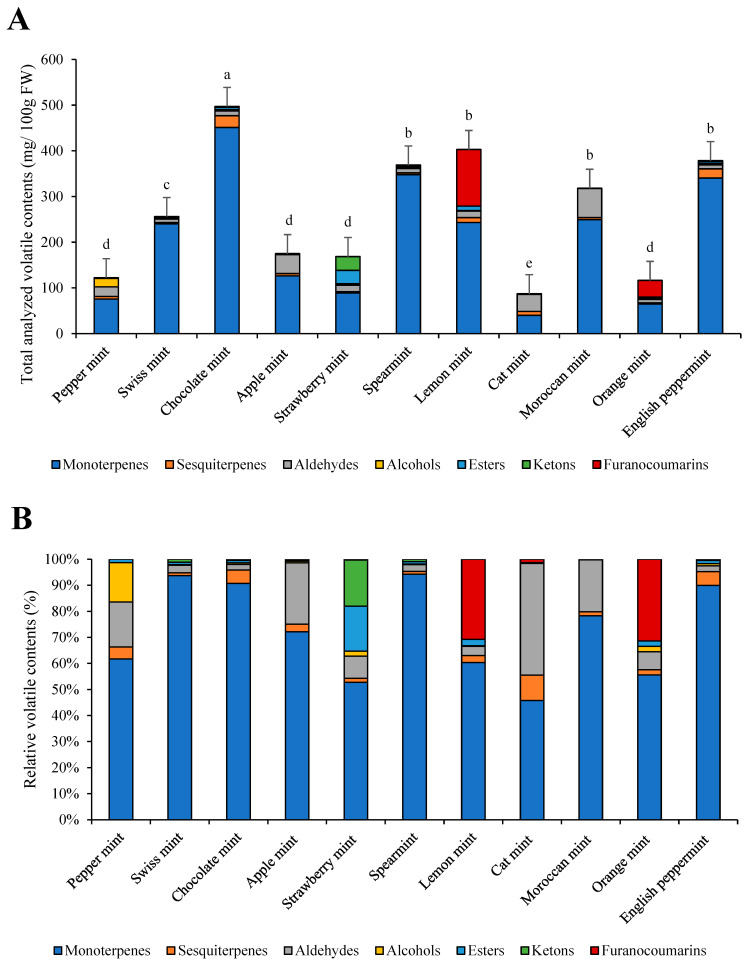
Individual volatile groups and total analyzed volatile contents in eleven mint cultivars (**A**) and their relative contents (**B**). a–e: statistical significant differences among different mint scented cultivars.

The most total volatiles were identified in chocolate mint, with 497.11 mg/100 g FW. The least volatiles were present in peppermint (122.41 mg/100 g FW), orange mint (116.44 mg/100 g FW), and cat mint (87.35 mg/100 g FW) (Figure 2A,B). The most abundant group of volatiles was monoterpenes, presenting from 45% (cat mint) up to 95% (Swiss mint) of the total volatile contents. In cat mint, apple mint, peppermint, and Moroccan mint, aldehydes presented from 20 to 40% of all volatiles. In strawberry mint, there were large amounts of ketones (around 15% of total volatiles). In lemon and orange mint, approximately 30% of all volatiles were presented by a furanocoumarin named bergamol.

The most abundant volatiles determined in the eleven mint species were *α*-pinene, ethyl 2-methylbutyrate, *β*-pinene, limonene, eucalyptol, *trans*-*β*-ocimene, *trans*-menthone, linalool, bergamol, carvone, and menthol (Table 3).

## 4. Discussion

The economically most relevant mint (mint-scented) cultivars cultivated in Slovenia were examined to assess their distinct phenolic and volatile compositions. This investigation aimed to identify the most favorable cultivar in terms of plant metabolites, making it suitable for consumption and widespread cultivation, particularly in the pharmaceutical industry. The eleven mint cultivars included peppermint, Swiss mint, chocolate mint, apple mint, strawberry mint, spearmint, lemon mint, Moroccan mint, cat mint, orange mint, and English peppermint.

We determined 19 individual phenolics and 80 individual volatiles, which had not been performed to such a large extent previously. The most common group of phenolics was flavones in all mint cultivars except for peppermint, where hydroxycinnamic acids were the most abundant. As reported by Pereira and Cardoso [28], eriocitrin, luteolin-7-*O*-rutinoside, luteolin-7-*O*-glucuronide, luteolin-7-*O*-glucoside, hesperidin, and diosmin are the most abundant phenolics in mints, most of which were also determined in our study, although they were not present in all cultivars. In peppermint, however, high concentrations of phenolic acids were determined. As reported by Bodalska, et al. [29], caffeic acid and its derivatives and rosmarinic acid have an important role in anti-inflammatory, antioxidant, and free-radical scavenging properties. Rosmarinic acid is especially reported to have excellent antimicrobial and antiviral activity [29].

Other mints had high luteolin concentrations. Luteolin and its variations have been reported to have antioxidative, antitumor, anti-inflammatory, and cardio-protective properties [30]. Swiss mint and spearmint had a high percentage of flavanones, especially eriocitrin, which is usually the dominant phenol in citrus fruits. Similar high eriocitrin contents were previously reported by Brown, John, and Shahidi [13] in mints. Flavanones are very effective against allergies, tumorigenesis, oxidative stress, aging-associated diseases, viral or bacterial diseases, and inflammatory issues [31].

Mints are well-known species of plants, especially due to their versatile and high volatile contents, which are their main characteristics. The most volatiles we determined were from the group of monoterpenes, as also reported by Park, et al. [8]. As reported by Cheallaigh, et al. [32], monoterpenoids are secondary metabolites that are not vital for the plant’s metabolic functioning. They are mostly synthesized by the plant to fend off predators. The most abundant monoterpenoids are menthol, camphor, limonene, and carvone, which were also determined in our mint cultivars [10]. In our study, we determined that Swiss mint and spearmint have the greatest amount of monoterpenoids, which suggests that these cultivars could be the target for genetic and agronomic improvement, obtaining high volatile yield while decreasing the need for large amounts of agricultural land. Mint plants (dry or fresh) are greatly used as a source of volatiles for confectionaries, flavor-enhancing agents in toothpastes, chewing gums, and beverages, bakeries, cosmetics, oral hygiene products, pharmaceuticals, and pesticides [3].

Other mint cultivars varied greatly in terms of their volatile profiles and contents. Due to the intricate biochemistry and molecular biology underlying plant volatiles, there are several hundred genes and pathways that play a crucial role in plant volatile synthesis and are responsible for the variable profiles of mints and other herbs [15]. The variability of volatiles and the perception of the aroma also change with the number of volatiles, their chemical nature, and the synergic effects, resulting in mints with so many different aromas [33].

With the results of our study, we can suggest the most volatile and phenolic-rich cultivars for wider production. In addition, by choosing metabolite-rich cultivars, the need for agricultural land decreases as we cover our needs with higher-yielding mints, as reported by Schneider, et al. [34]. With reduced land use, the pressure on the ecosystem is also reduced.

## 5. Conclusions

In our study, we analyzed 11 of the most common mint cultivars in Slovenia, determining 19 individual phenolics and 80 individual volatiles. This study provides a crucial understanding of optimal mint cultivars and their potential for wider cultivation due to their phenolic and volatile contents. Notably, chocolate mint demonstrated significant potential in terms of volatiles, while apple mint showed promise in phenolics. These cultivars hold promise for intense production across agricultural, pharmaceutical, and food industries, as they could increase metabolite yields with targeted cultivation, reducing the demand for agricultural land, which would lean toward environmentally friendly production. Additionally, consuming mint cultivars rich in phenolics and volatiles may lead to improved health-promoting effects in humans.

## Figures and Tables

**Table 1:** Tentative identification of the 19 phenolics from 11 different mint cultivars.

Compound	Rt (min)	[M−H]^−^ (*m*/*z*)	MS^2^ (*m*/*z*)	MS^3^ (*m*/*z*)	Orange Mint	Chocolate Mint	Peppermint	Spearmint	Apple Mint	Strawberry Mint	Swiss Mint	English Peppermint	Lemon Mint	Moroccan Mint	Cat Mint
Protocatechuic acid	7.71	315	153 (100), 123 (5)												X
Rosmarinic acid	15.14	385	**161** (100), 179 (20), 197 (18), 223(5)	133 (100)			X								
Caffeic acid	15.46	179	161 (100), 135 (75)				X		X						
Luteolin-di-3-glucoside	18.79	813	**637** (100), 461 (17), 351 (13), 285 (10)	351 (100), 285 (29)		X			X				X		
Eriocitrin	19.09	595.5	287 (100)		X	X		X	X		X	X	X	X	
Luteolin derivative 1	19.61	623	461 (100), 447 (31), 285 (20), 501 (20)		X	X		X		X	X	X			
Luteolin rutinoside	20.08	593	285 (100)		X	X		X	X	X	X	X	X	X	
Salvianolic acid	20.63	537	339 (100), 493 (16)				X					X			
Salvianolic acid A isomer	20.82	403	223 (100), 357 (68), 179 (18)				X								
Luteolin hexoside	21.11	447	285 (100)							X				X	
Luteolin derivative 2	21.87	623	579 (100), 535 (37), 285 (21), 315 (20)		X				X		X		X		
Naringenin-7-*O*-rutinoside	21.92	579	269 (100), 270 (97), 271 (40), 245 (21)			X	X		X		X	X			
Lithosperimic acid derivative	22.28	655	359 (100), 295 (31), 637 (37)				X	X		X	X		X	X	
Diosmin	22.36	607.5	299 (100), 284 (44)				X								
Luteolin-*O*-glucuronide	22.37	461	285 (100)		X	X			X	X			X		X
Salvianolic acid B isomer	23.57	717	519 (100), 475 (24), 339 (9)				X								
Rosmarinic acid derivative	24.47	359	161 (100), 179 (26), 197 (23), 223 (14)				X		X						X
Sagerinic acid	24.55	719	**557** (100), 359 (30)	359 (100), 161 (80), 179 (29), 197 (23), 223 (15)										X	
Salvianolic acid B/E isomer	25.33	717	519 (100), 537 (52), 673 (43), 493 (7)				X								

Rt = retention time; [M−H]^−^ = main mass of substance in negative ion mode; MS^2^ = first fragmentation; MS^3^ = second fragmentation. X = phenolic compound identified in mint cultivar.

**Table 2:** Individual phenolic content in 11 mint cultivars.

Phenolics	Orange Mint	Chocolate Mint	Peppermint	Spearmint	Apple Mint	Strawberry Mint	Swiss Mint	English Peppermint	Lemon Mint	Moroccan Mint	Cat Mint
Rosmarinic acid			35.36 ± 4.58								
Caffeic acid			178.57 ± 40.84		60.61 ± 3.87					122.50 ± 1.49	
Salvianolic acid			76.80 ± 22.46					129.34 ± 34.99			
Salvianolic acid A isomer			283.47 ± 87.59								
Naringenin-7-*O*-rutinoside		22.84 ± 7.30	49.95 ± 5.30		10.33 ± 0.81		86.85 ± 17.55	20.34 ± 4.53			
Lithosperimic acid derivative			312.41 ± 55.87	149.26 ± 20.28		1129.02 ± 136.50	348.72 ± 43.55		271.40 ± 40.72	299.44 ± 7.23	
Diosmin			51.65 ± 15.69								
Salvianolic acid B isomer			208.09 ± 57.29								
Rosmarinic acid derivative			216.54 ± 77.37		126.21 ± 32.41						14.41 ± 0.59
Salvianolic acid B/E isomer			125.68 ± 4.78							36.00 ± 5.01	
Eriocitrin	50.46 ± 7.77	171.46 ± 19.36		857.66 ± 64.60	332.08 ± 30.43		420.37 ± 148.68	137.42 ± 34.22	175.16 ± 36.78	113.86 ± 2.15	
Luteolin derivative 1	254.58 ± 52.00	120.27 ± 21.14		874.66 ± 56.41		109.20 ± 6.98	125.49 ± 7.71	218.75 ± 23.22			
Luteolin rutinoside	215.10 ± 35.48	1127.07 ± 64.88		1238.63 ± 55.74	1800.21 ± 163.53	91.60 ± 11.22	1162.34 ± 208.59	730.89 ± 143.31	642.15 ± 296.32	981.67 ± 28.11	
Luteolin derivative 2	524.87 ± 94.73				352.61 ± 35.21		382.60 ± 136.24		272.48 ± 31.94		
Luteolin-di-3-glucoside		78.56 ± 8.95		109.60 ± 20.80					148.23 ± 20.05		
Luteolin-*O*-glucuronide	43.55 ± 8.58	863.77 ± 219.81			656.88 ± 60.87	313.08 ± 19.29			215.28 ± 15.58		459.18 ± 55.64
Luteolin hexoside						116.67 ± 19.05				92.07 ± 36.41	
Protocatechuic acid											86.25 ± 7.34
Sagerinic acid										607.52 ± 18.80	

**Table 3:** Individual contents of volatiles in 11 mint cultivars.

Volatile	Orange Mint	Chocolate Mint	Pepper Mint	Spearmint	Apple Mint	Strawberry Mint	Swiss Mint	English Peppermint	Lemon Mint	Moroccan Mint	Cat Mint
*α*-Pinene	1.24 ± 0.07	21.15 ± 2.96	9.80 ± 1.26	13.06 ± 1.15	6.64 ± 0.25	16.74 ± 2.34	7.86 ± 0.38	15.06 ± 1.85	7.64 ± 0.02	11.81 ± 1.17	2.91 ± 0.05
*α*-Thujene		0.95 ± 0.13	0.48 ± 0.06	0.67 ± 0.06	0.16 ± 0.01	0.24 ± 0.09	0.35 ± 0.01	0.89 ± 0.10	0.34 ± 0.01	0.40 ± 0.04	0.49 ± 0.02
Ethyl 2-methylbutyrate		0.56 ± 0.06	0.76 ± 0.13	1.41 ± 0.10	0.38 ± 0.01	29.03 ± 5.81	0.52 ± 0.03	0.30 ± 0.03		0.28 ± 0.03	
Camphene		0.42 ± 0.06	6.19 ± 0.74	0.33 ± 0.03	0.16 ± 0.01	2.99 ± 0.39	0.19 ± 0.01	0.64 ± 0.09		0.21 ± 0.02	
Hexanal	4.13 ± 0.24	5.23 ± 0.49	4.18 ± 1.01	2.92 ± 0.37	2.04 ± 0.15	1.68 ± 0.46	2.47 ± 0.18	3.94 ± 0.23	9.30 ± 0.57	2.43 ± 0.34	1.83 ± 0.07
*β*-Pinene	2.36 ± 0.12	24.48 ± 3.42	16.11 ± 1.83	15.27 ± 1.41	6.12 ± 0.24	12.39 ± 1.48	9.18 ± 0.31	17.83 ± 2.37	13.74 ± 0.02	12.16 ± 1.19	10.13 ± 0.15
Sabinene	1.57 ± 0.06	14.56 ± 2.06	3.58 ± 0.39	11.10 ± 1.14	4.30 ± 0.18	5.22 ± 0.64	6.41 ± 0.14	10.48 ± 1.48	9.45 ± 0.07	8.63 ± 0.84	3.07 ± 0.05
Myrcene	2.45 ± 0.05	6.73 ± 0.98	3.48 ± 0.36	6.76 ± 0.78	4.52 ± 0.23	3.75 ± 0.43	3.84 ± 0.04	5.02 ± 0.73	10.90 ± 0.36	18.20 ± 1.73	1.03 ± 0.02
*p*-mentha-1(7),8-diene			0.02 ± 0.02	0.24 ± 0.02	0.21 ± 0.01	0.14 ± 0.02				0.22 ± 0.03	
Limonene	0.76 ± 0.02	32.20 ± 4.31	19.54 ± 1.85	64.47 ± 6.19	83.79 ± 2.94	24.34 ± 2.74	45.09 ± 0.88	22.84 ± 2.94	4.18 ± 0.07	150.61 ± 11.08	1.04 ± 0.11
*α*-Phellandrene			0.08 ± 0.01		0.25 ± 0.02	0.52 ± 0.04		0.23 ± 0.02		0.58 ± 0.04	1.95 ± 0.04
Eucalyptol	18.16 ± 1.19	88.08 ± 8.80	12.40 ± 1.87	57.14 ± 4.31	18.40 ± 0.95	1.11 ± 0.18	36.33 ± 0.92	69.90 ± 4.71	71.11 ± 0.59	36.55 ± 2.05	10.81 ± 0.20
*p*-Mentha-1,3,8-triene										0.21 ± 0.11	
*trans*-2-Hexenal	3.56 ± 0.11	3.75 ± 0.14	6.05 ± 1.47	4.34 ± 0.29	7.16 ± 0.32	11.79 ± 3.80	4.33 ± 0.10	2.96 ± 0.29	4.76 ± 0.36	6.63 ± 0.98	1.10 ± 0.03
*trans*-*β*-Ocimene	1.23 ± 0.01	9.34 ± 1.94	0.88 ± 0.03	11.58 ± 2.25	1.00 ± 0.14	16.84 ± 0.85	5.53 ± 0.47	5.29 ± 1.51	44.08 ± 1.24	5.34 ± 0.52	2.13 ± 0.04
*γ*-Terpinene		0.21 ± 0.03		0.06 ± 0.01				0.22 ± 0.02	1.93 ± 0.26		
*β*-Ocimene	1.05 ± 0.02	1.07 ± 0.35	0.29 ± 0.03	1.96 ± 0.76	0.15 ± 0.05	0.31 ± 0.01	0.92 ± 0.21	0.77 ± 0.38	10.55 ± 0.59	1.17 ± 0.17	6.16 ± 0.12
3-Octanone			0.70 ± 0.10								0.93 ± 0.01
Styrene											0.87 ± 0.01
*p*-Cymene	0.35 ± 0.04	0.81 ± 0.08	0.05 ± 0.01	0.63 ± 0.03		0.06 ± 0.01	0.48 ± 0.03	0.97 ± 0.06	3.34 ± 0.35		
2-Methylbutyl 2-methylbutanoate		1.27 ± 0.16	0.34 ± 0.03	0.44 ± 0.04		0.10 ± 0.04	0.27 ± 0.01	0.92 ± 0.06	2.51 ± 0.01	0.15 ± 0.01	
Terpinolene	0.09 ± 0.02	0.19 ± 0.07	0.07 ± 0.04			0.32 ± 0.01		0.19 ± 0.09	0.33 ± 0.01	0.27 ± 0.04	
4-Carene											
2-Methylbutyl isovalerate		0.65 ± 0.13	0.16 ± 0.01	0.43 ± 0.05			0.62 ± 0.02	0.60 ± 0.16	1.61 ± 0.01		
3-Heptanol			0.17 ± 0.03			0.57 ± 0.16					
*trans*-allo-Ocimene		0.28 ± 0.05		0.35 ± 0.07		0.55 ± 0.05	0.17 ± 0.01	0.15 ± 0.05	1.48 ± 0.04	0.14 ± 0.01	
1-Octen-3-yl-acetate	1.01 ± 0.13								1.01 ± 0.06		
3-Hexenol			0.15 ± 0.05			0.19 ± 0.05					
3-Octanol	0.81 ± 0.14	1.59 ± 0.10	18.19 ± 2.60	1.03 ± 0.05	1.06 ± 0.08		0.48 ± 0.01	2.12 ± 0.20	0.29 ± 0.03		
2,4-Hexadienal	0.09 ± 0.02	0.31 ± 0.03	0.13 ± 0.02	0.16 ± 0.02	0.11 ± 0.01	0.17 ± 0.04	0.11 ± 0.01	0.12 ± 0.07	0.32 ± 0.01		
*p*-Cymenene			0.05 ± 0.01		0.15 ± 0.01	0.06 ± 0.01				0.21 ± 0.03	
*cis*-Limonene oxide			0.17 ± 0.10	0.66 ± 0.08	1.41 ± 0.09		0.45 ± 0.03	0.24 ± 0.02		2.00 ± 0.19	
3-Octenol	1.45 ± 0.19	0.96 ± 0.06				2.57 ± 0.56		0.57 ± 0.07			0.15 ± 0.01
Vaporole									0.69 ± 0.09		
*trans*-Limonene oxide			0.15 ± 0.10							0.17 ± 0.01	
*trans*-Menthone	0.15 ± 0.04	174.69 ± 16.84	1.62 ± 0.19	123.18 ± 7.88	0.41 ± 0.08	0.60 ± 0.16	87.04 ± 2.15	134.34 ± 13.86	2.94 ± 0.13		0.57 ± 0.01
Sabinene hydrate										2.57 ± 0.13	
*cis*-Linalool oxide	0.08 ± 0.01		0.16 ± 0.09						0.51 ± 0.02		
*cis*-3-hexenyl-Isovalerate										0.17 ± 0.02	
Copaene											0.13 ± 0.01
*cis*-Menthone		25.16 ± 2.65	0.07 ± 0.02	9.49 ± 0.60			6.73 ± 0.15	16.43 ± 1.61	0.24 ± 0.01		
*β*-Bourbonene		0.45 ± 0.05	0.35 ± 0.03	0.24 ± 0.04	0.73 ± 0.05	0.10 ± 0.01	0.26 ± 0.02	0.40 ± 0.07		0.78 ± 0.07	0.18 ± 0.01
Neomenthyl acetate								0.20 ± 0.04			
*α*-Gurjunene	0.13 ± 0.01						0.24 ± 0.02		0.39 ± 0.02		
Linalool	35.02 ± 2.02	1.54 ± 0.18	0.05 ± 0.00	0.43 ± 0.03		3.03 ± 0.55	0.24 ± 0.01	0.50 ± 0.09	58.83 ± 1.24	0.26 ± 0.02	0.59 ± 0.02
*cis*-Pinocamphone				0.28 ± 0.01							
Bergamol	36.57 ± 1.24					0.47 ± 0.07		0.28 ± 0.06	123.95 ± 2.43		1.14 ± 0.09
Menthyl acetate		2.23 ± 0.27		1.11 ± 0.15			1.07 ± 0.03	2.39 ± 0.48			
Isopulegol		0.50 ± 0.05						0.37 ± 0.03			
Bornyl acetate			0.29 ± 0.01								
β-Elemene				0.14 ± 0.01				0.18 ± 0.02			
β-Copaene											
β-Caryophyllene	0.08 ± 0.01	16.33 ± 1.80	2.39 ± 0.22		1.80 ± 0.11	0.09 ± 0.01		13.08 ± 2.22	4.94 ± 0.19	1.73 ± 0.16	1.36 ± 0.10
Isoneomenthol				3.71 ± 0.39			4.06 ± 0.03				
Lavandulyl acetate	0.21 ± 0.01								1.50 ± 0.12		
*cis*-Dihydrocarvone			0.15 ± 0.02		0.66 ± 0.02					0.33 ± 0.02	
Myrtenal						0.39 ± 0.07					
Menthol		48.62 ± 5.73	0.34 ± 0.04	26.98 ± 2.34	0.10 ± 0.01	0.19 ± 0.03	25.56 ± 0.83	38.29 ± 5.43	0.70 ± 0.05		0.18 ± 0.01
Pulegone		0.43 ± 0.06		1.03 ± 0.10				0.62 ± 0.10		0.78 ± 0.05	
*trans*-*β*-Farnesene	0.34 ± 0.01	1.05 ± 0.11	0.16 ± 0.01	0.82 ± 0.14	0.27 ± 0.01		0.45 ± 0.04	0.75 ± 0.12	0.74 ± 0.05	0.30 ± 0.02	0.45 ± 0.04
*α*-Humulene		0.41 ± 0.05	0.09 ± 0.01		0.14 ± 0.01			0.32 ± 0.03	0.32 ± 0.02	0.32 ± 0.03	
*δ*-Terpineol		0.32 ± 0.04		0.21 ± 0.01			0.17 ± 0.01	0.42 ± 0.02			
Lavandulol	0.20 ± 0.02							0.18 ± 0.02	0.60 ± 0.05		
*α*-Terpineol	0.19 ± 0.05	0.26 ± 0.03	0.21 ± 0.03	0.17 ± 0.01	0.25 ± 0.02	0.07 ± 0.01		0.24 ± 0.06	0.73 ± 0.02		
Borneol			1.62 ± 0.20			0.62 ± 0.13					
Germacrene D	1.13 ± 0.05	7.47 ± 0.88	1.06 ± 0.09	2.74 ± 0.60	2.17 ± 0.16	1.85 ± 0.07	1.91 ± 0.24	5.35 ± 1.07	2.98 ± 0.17	1.62 ± 0.15	6.13 ± 0.56
*α*-Funebrene											0.12 ± 0.01
*cis*-Geranyl acetate	0.51 ± 0.02								1.48 ± 0.10		
*β*-Bisabolene											0.12 ± 0.01
Geranial	0.09 ± 0.01										
Piperitone		2.21 ± 0.27		2.98 ± 0.31	0.91 ± 0.07		2.41 ± 0.03	1.54 ± 0.20			
Carvone	0.22 ± 0.03	0.66 ± 0.14	10.48 ± 1.03	0.47 ± 0.15	29.94 ± 2.12	0.22 ± 0.08	0.12 ± 0.03	0.71 ± 0.14		51.40 ± 2.69	0.18 ± 0.12
*trans*-Geranyl acetate	1.14 ± 0.04								3.19 ± 0.22		0.10 ± 0.00
*p*-Mentha-1,8-dien-3-one						1.71 ± 0.25					
Geraniol											
Piperitenone						7.64 ± 1.04	0.19 ± 0.08				
*cis*-Jasmone						0.14 ± 0.02					
Piperitenone oxide						20.39 ± 2.68	0.25 ± 0.10				
Nepetalactone 1											33.72 ± 3.40
Nepetalactone 2											0.67 ± 0.06

## Data Availability

The data presented in this study are available on request from the corresponding author. The data are not publicly available due to privacy restrictions.
